# Effects of bariatric surgery on breast density in adult obese women: systematic review and meta-analysis

**DOI:** 10.3389/fimmu.2023.1160809

**Published:** 2023-05-31

**Authors:** Dezheng Sun, Zhiping Huang, Wenyan Dong, Xiang Zhao, Chaoqian Liu, Yuan Sheng

**Affiliations:** ^1^ Department of Thyroid and Breast Surgery, Changhai Hospital Affiliated to Naval Medical University, Shanghai, China; ^2^ Department of Hepatobiliary Surgery and Organ Transplantation, General Hospital of Southern Theater Command of People's Liberation Army of China (PLA), Guangzhou, China

**Keywords:** bariatric surgery, breast density, obesity, mammogram, meta-analysis

## Abstract

**Introduction:**

Bariatric surgery is one of the most effective methods for treating obesity. It can effectively reduce body weight and reduce the incidence of obesity-related breast cancer. However, there are different conclusions about how bariatric surgery changes breast density. The purpose of this study was to clarify the changes in breast density from before to after bariatric surgery.

**Methods:**

The relevant literature was searched through PubMed and Embase to screen for studies. Meta-analysis was used to clarify the changes in breast density from before to after bariatric surgery.

**Results:**

A total of seven studies were included in this systematic review and meta-analysis, including a total of 535 people. The average body mass index decreased from 45.3 kg/m^2^ before surgery to 34.4 kg/m^2^ after surgery. By the Breast Imaging Reporting and Data System score, the proportion of grade A breast density from before to after bariatric surgery decreased by 3.83% (183 vs. 176), grade B (248 vs. 263) increased by 6.05%, grade C (94 vs. 89) decreased by 5.32%, and grade D (1 vs. 4) increased by 300%. There was no significant change in breast density from before to after bariatric surgery (OR=1.27, 95% confidence interval (CI) [0.74, 2.20], P=0.38). By the Volpara density grade score, postoperative volumetric breast density increased (standardized mean difference = -0.68, 95% CI [-1.08, -0.27], P = 0.001).

**Discussions:**

Breast density increased significantly after bariatric surgery, but this depended on the method of detecting breast density. Further randomized controlled studies are needed to validate our conclusions.

## Introduction

Obesity is a chronic metabolic disease whose global prevalence is rapidly increasing (1). The American Institute for Cancer Research found that obesity was linked to an increased risk of 13 types of cancers (2). Bariatric surgery is currently one of the most effective treatments for obesity, which can reduce body weight by an average of at least 15% (3). It is effective at reducing weight while also reducing the risk of obesity-related cancers, especially postmenopausal breast cancer, endometrial cancer, and colon cancer (4).

Breast density is an indicator of the number of dense and nondense areas in the breast and can be expressed as the ratio of dense areas to the total breast area (5). At present, breast density can be assessed as qualitative breast density and quantitative breast density. Qualitative breast density is primarily assessed by the Breast Imaging Reporting and Data System (BI-RADS) scoring system (6). Quantitative breast density is measured by mammography computer-aided detection (CAD) diagnostic system software, such as the Laboratory for Individualized Breast Radiodensity Assessment (LIBRA), Quantra, and Volpara (7–9). Volpara software uses fibroglandular volume, total breast volume, and the ratio of the two, called volumetric breast density (VBD), to quantitatively assess breast density (9). Volpara’s quantitative breast density score, Volpara density grade (VDG), can be translated to a BI-RADS score as follows: VDG 1: VBD<4.5%; VDG 2: 4.5%≤VBD<7.5%; VDG 3: 7.5%≤VBD ≤ 15.5%; and VDG 4: VBD>15.5%.

Although bariatric surgery can reduce the risk of breast cancer (4), there are inconsistent conclusions about the changes in breast density from before to after bariatric surgery. In this study, we searched the published literature to investigate changes in breast density from before to after bariatric surgery by means of systematic review and meta-analysis.

## Methods

### Data sources and search strategy

A systematic search of literature on changes in breast density from before to after bariatric surgery was conducted in PubMed and Embase. The last search was on 6 April 2023. Key search terms included “bariatric surgery”, “Sleeve gastrectomy”, “Roux-en-Y”, “ Gastric banding”, and “breast”. The search statements were refined by finding synonyms and hyponyms for these key search terms (**Supplementary Material 1**).

This study was conducted in accordance with the Preferred Reporting Items for Systematic Reviews and Meta-Analyses (PRISMA) guidelines from 2020 (**Supplementary Material 2**).

### Eligibility criteria

Inclusion criteria: (1) follow-up > 1 year, (2) P: adult obese female, (3) I: bariatric surgery for weight loss, (4) sample size ≥ 10, (5) C: preoperative and postoperative mammography, (6)O: changes in breast density, and (7)S: cohort study. Exclusion criteria: (1) lack of preoperative or postoperative mammography, (2) subjects younger than 18 years, (3) combination of bariatric surgery and other interventions for weight loss, and(4)conference abstract.

### Study selection

After importing the retrieved literature into Note Express 3.7.0.9296, duplicate studies were removed, and articles with topics irrelevant to this study were removed by reading the title and abstract. Then, by reading the full text, two independent reviewers (Dezheng Sun and Zhiping Huang) further screened the remaining papers based on the inclusion and exclusion criteria. In case of disagreement, consensus was reached through discussion. If disagreements persisted, an independent reviewer (Chaoqian Liu) made the final decision.

### Data extraction and quality assessment

The following information was extracted from the included papers: definition of breast density, method of evaluating breast density, evaluation index of breast density, type of bariatric surgery, type of study, sample size of study, preoperative and postoperative body mass index (BMI), preoperative follow-up time from preoperative X-ray to postoperative X-ray, number of cases of each grade of breast density, publication year, country, and number of citations.The quality of the literature was evaluated according to the Newcastle−Ottawa scale (10), which was scored from the three aspects of selection of cohort studies, comparability, and outcomes. Each article can be given up to nine stars: 7-9 stars are high-quality articles, 4-6 stars are medium-quality articles, and ≤3 stars are low-quality articles.

### Statistical analysis

The effect of bariatric surgery on breast density was comprehensively evaluated by the OR value, SMD value, and P value, and the results are displayed in forest plots. Heterogeneity of studies was assessed by the I² value and Cochran Q test. In the Cochran Q test, P<0.05 indicated significant heterogeneity. By I², there was heterogeneity when I²>50% (11). If there was a contradiction between the two, I² was the priority criterion for heterogeneity evaluation. If there was heterogeneity, sensitivity analysis and meta-regression analysis were performed to explore the source of the heterogeneity. In addition, publication bias was evaluated by funnel plot and eggers test. When conducting data analysis, the median can be used to estimate the mean when the sample size of the study is greater than 25. If the sample size of the study is large and the data distribution is close to a normal distribution, the interquartile range is approximately equal to 1.35 times the standard deviation (12). Ratios were compared by Fisher’s exact method. Statistical analysis was performed in Review Manager 5.4, IBM SPSS Statistics 26 and Stata14.

## Results

### Study retrieval

**Figure 1:**
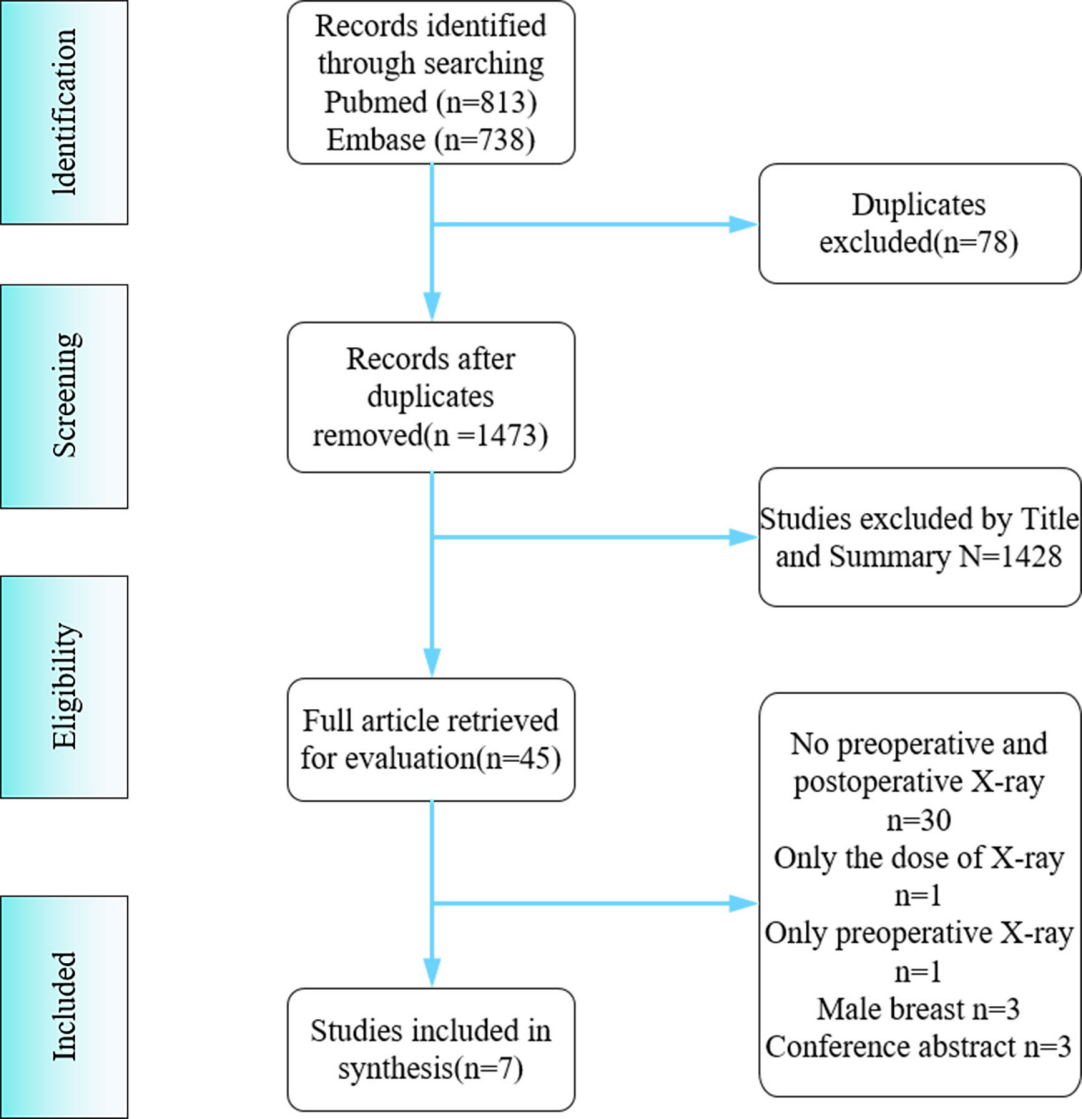
Flow chart of the literature search.

A total of 1551 documents were retrieved from PubMed search (N=813) and Embase search (N=738). After screening, 78 duplicates were deleted, 1428 were excluded by reading titles and abstracts, and 38 were excluded after full-text reading, leaving seven studies for this systematic review and meta-analysis (**Supplementary Material 1**). The reasons for exclusion and the screening process are shown in **Figure 1**.

### Study characteristics

All seven studies were retrospective studies. They included a total of 535 people. Among them, 302 (56.4%) received Roux-en-Y gastric bypass, 196 (36.6%) received sleeve gastrectomy, and 37 (6.9%) received laparoscopic adjustable gastric banding. The mean BMI decreased from 45.3 kg/m^2^ preoperatively to 34.4 kg/m^2^ after surgery. The mean follow-up period was 3.5 years. All seven studies used the BI-RADS scale (6, 13–19). Four (13, 15, 17, 19) used two density scoring methods and evaluation criteria (**Table 1**). When we compared the BI-RADS scores before and after bariatric surgery (**Table 2**), we found the following changes: grade A: 183 vs. 176; grade B: 248 vs. 263; grade C: 94 vs. 89; and grade D: 1 vs. 4. BI-RADS grade B was the most common density type, being found in 248 (47.1%) patients preoperatively and 263 (49.4%) patients postoperatively (P=0.509).

**Table 1:** Study characteristics of included studies

Study	Study design	Reference	Country	Sample size				Mean Preoperative BMI(kg/m2)	Mean Postoperative BMI(kg/m2)	Mean time from preoperative mammogram to postoperative mammogram (year)	Assessment methods of Breast Density	Study outcomes	Source of funds
				RYGB	SG	LAGB	Total						
Taryn et al. (13)	Retrospective	38	USA	137	24	19	180	46	35.4	5.925	BI-RADS, Volpara	breast volume,VBD,FGV, BI-RADS scores	the National Institutes of Health surgical oncology grant [T32 CA163177]
Ava et al. (14)	Retrospective	18	USA	29	13	0	42	43.8	30.3	1.90	BI-RADS	BI-RADS scores	None
Rafael et al. (15)	Retrospective	29	USA	0	50	0	50	46.2	35.7	2.535	BI-RADS, software	BI-RADS scores, BA,BD, ADA	T32(1T32DK108740),P30 (DK089503) , R01 (DK107652)
Natalia et al. (16)	Retrospective	32	USA	7	46	10	63	44.3	33.7	2.7	BI-RADS	BI-RADS scores, MD	None
Austin et al. (17)	Retrospective	30	USA	59	51	0	110	44.8	34.9	2.46	BI-RADS, LIBRA	BI-RADS scores ,BA,DA, BD, PD	None
Tara et al. (18)	Retrospective	21	USA	5	4	1	10	42.3	29.2	1.32	BI-RADS	BI-RADS scores, breast thickness PNL, mAs, kVp,	None
Nasreen et al. (19)	Retrospective	50	USA	65	8	7	80	46	33.7	1.81	BI-RADS, Volpara	breast volume,VBD FGV, BI-RADS scores	None

LAGB, laparoscopic adjustable gastric banding; RYGB, Roux-en-Y gastric bypass; BI-RADS, Breast Imaging Reporting and Data System scores; VBD, volumetric breast density ; FGV, fibroglandular volume; SG, sleeve gastrectomy; BMI , body mass index; BA, breast area; BD, breast density; ADA, absolute dense breast area; MD, mammographic density; LIBRA ,the Laboratory for Individualized Breast Radiodensity Assessment; PD, percent density; PNL, pectoral nipple line; kVp, kilovoltage ; mAs, miliamperes per second; DA, dense area;

**Table 2:** Change of BI-RADS density level of Mammary glands.

BI-RADS density level (%)	Preoperative	Postoperative	P value*
a	183(34.8)	176(33.1)	
b	248(47.1)	263(49.4)	
c	94(17.9)	89(16.7)	
d	1 (0.2)	4(0.8)	
Total	526(100)	532(100)	0.509

* Calculated by Fisher’s Exact Test.

In Alvarez et al. (2018) study, the number of people in BI-RADS B and C were counted together. We processed the data by dividing the population of B+C equally between B and C. In Mokhtari et al. (2017) study, the left and right breast density grades were calculated separately, and we divided them by 2 when conducting data analysis.

### Quality assessment

In **Table 3**, the seven included articles are evaluated according to the Newcastle−Ottawa Scale (10). Five were high-quality studies and two were medium-quality. The specific scoring points were set as follows: In the selection of cohort studies, we decided that the exposure cohort was representative when the sample size was >100. In terms of comparability, we considered that the most important variables could be controlled for only if the effect of different surgical modalities on breast density was considered or if only one surgical modality was used. In terms of outcomes, we considered ≥1 year and ≤3 years of postoperative follow-up was adequate.

**Table 3:** Quality assessment of eligible studies.

Study	Selection	Comparability	Outcomes/exposure	Total
Taryn E. Hassinger 2019	★★★★	★	★★	7
Ava Hosseini 2019	★★★	★★	★★★	8
Rafael Alvarez 2018	★★★	★★	★★★	8
Natalia Partain 2018	★★★	★	★★★	7
Austin D.Williams 2017	★★★★	★	★★★	8
Tara E. Mokhtari 2017	★★★	★	★★	6
Nasreen A. Vohra 2017	★★★	★	★★	6

### Meta-analysis of breast density by BI-RADS system

**Figure 2:**
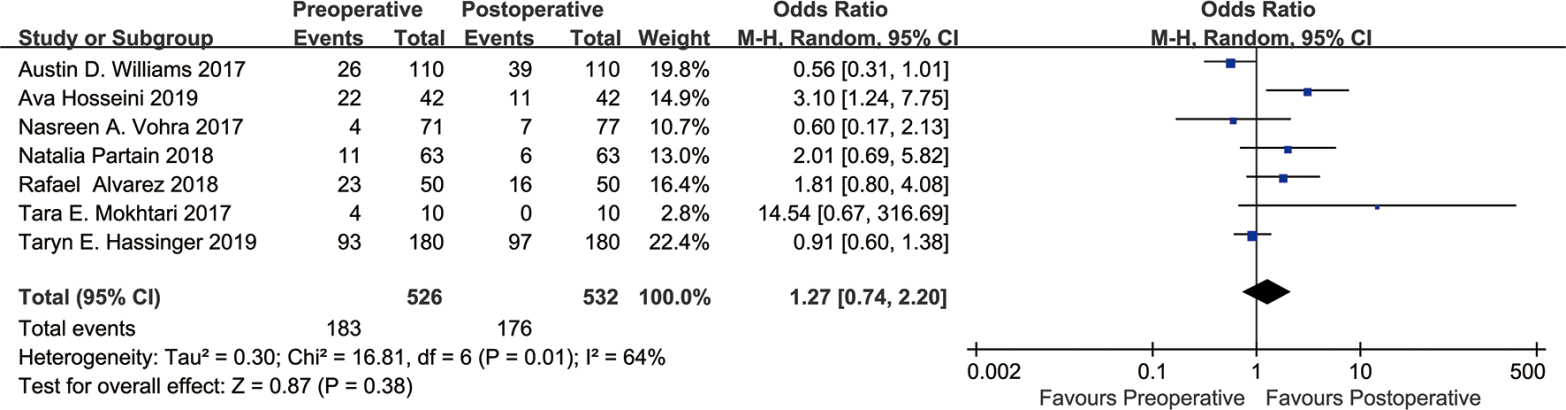
Forest plot of BI-RADS scores BI-RADS grade A was grouped into one category, and grades B, C, D were grouped into one category. Meta-analyses were performed according to dichotomous variables. Since grade D occurred only five times in the seven studies either before or after bariatric surgery, we tried grouping grade D with grades B, C into one group, and this had no significant effect on the results.

In this study, a meta-analysis was performed on the seven included studies (13–19) using the BI-RADS score as the evaluation standard. The analysis was conducted with dichotomous variables (**Figure 2**). The results showed that the density grade A from before to after bariatric surgery (183 vs. 176) decreased by 3.83%; heterogeneity: χ² = 16.81, I² = 64%, OR=1.27, 95% confidence interval (CI) [0.74, 2.20]. There was no significant change in breast density from before to after bariatric surgery (P=0.38).

### Meta-analysis of breast density by the VDG system

**Figure 3:**
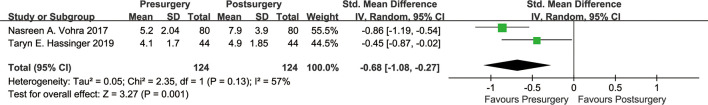
Forest plot of VDG score.

Two of the seven included studies used the VDG score (13, 19). We set the VDG score as a continuous variable and performed a meta-analysis of these two studies (**Figure 3**). The results revealed heterogeneity: χ² = 2.35, I² = 57%, SMD=-0.68, 95% CI [-1.08, -0.27]. According to the VDG score, VBD was significantly increased after bariatric surgery (P=0.001).

### Evaluation of heterogeneity and bias

**Figure 4:**
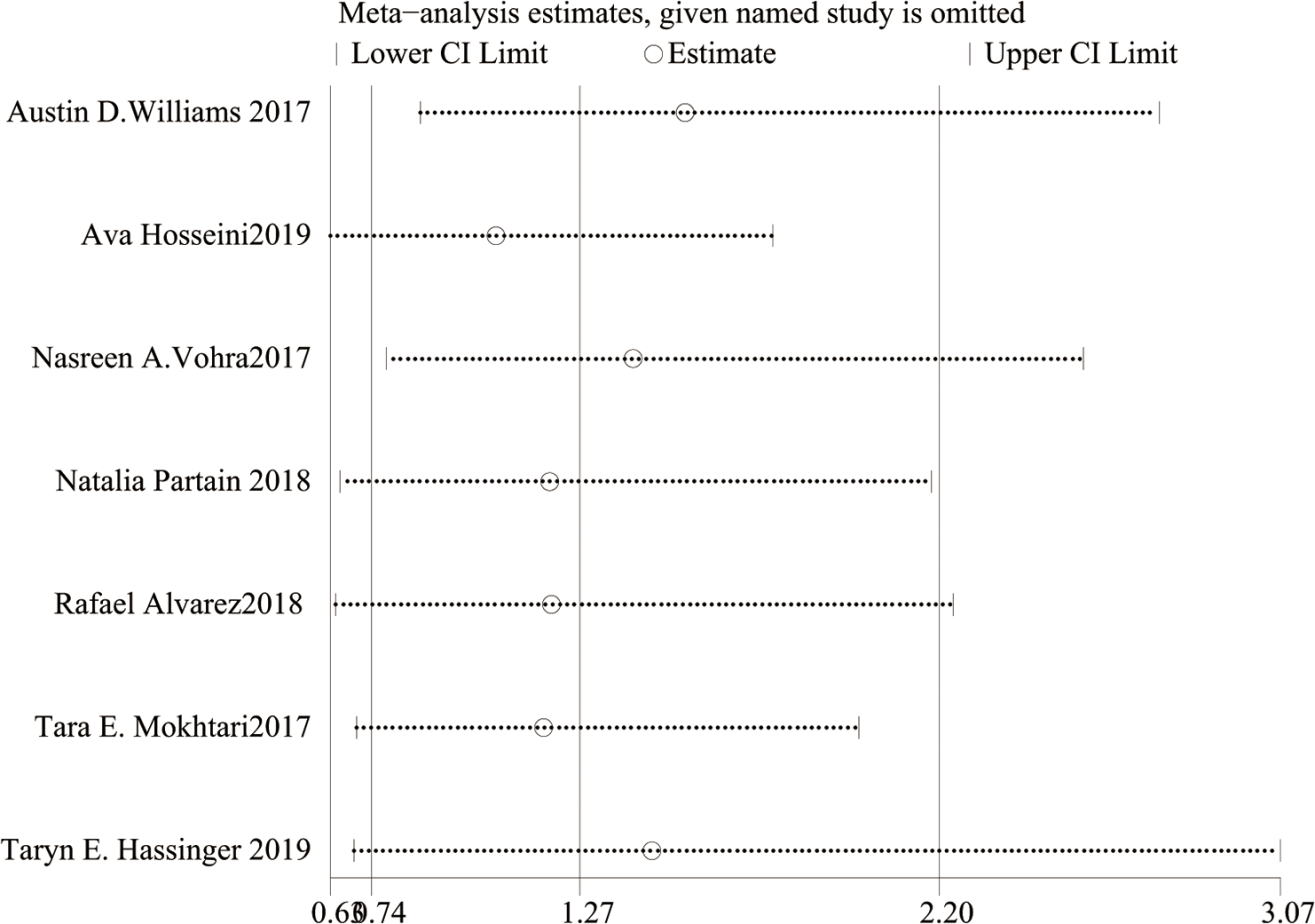
Sensitivity analysis plot of BI-RADS score.

As shown in **Figure 2**, there was heterogeneity between the studies (I²=64%, Cochran’s Q-test P=0.01), so a random-effect model was used for meta-analysis. Sensitivity analysis showed (**Figure 4**) that there was no significant heterogeneity in our study. In addition, we conducted regression analysis with the published time, type of surgery, follow-up time and BMI decline as covariables, and the results showed that RYGB (P=0.018) had a certain effect on heterogeneity, while other variables had no significant effect on heterogeneity (**Supplementary Material 1**). In **Figure 3**, I²=57%, Cochran Q test P=0.13, meaning there was heterogeneity, and a random-effect model was used. Since only two studies were included in **Figure 3**, regression analysis and sensitivity analysis were not performed.

**Figure 5:**
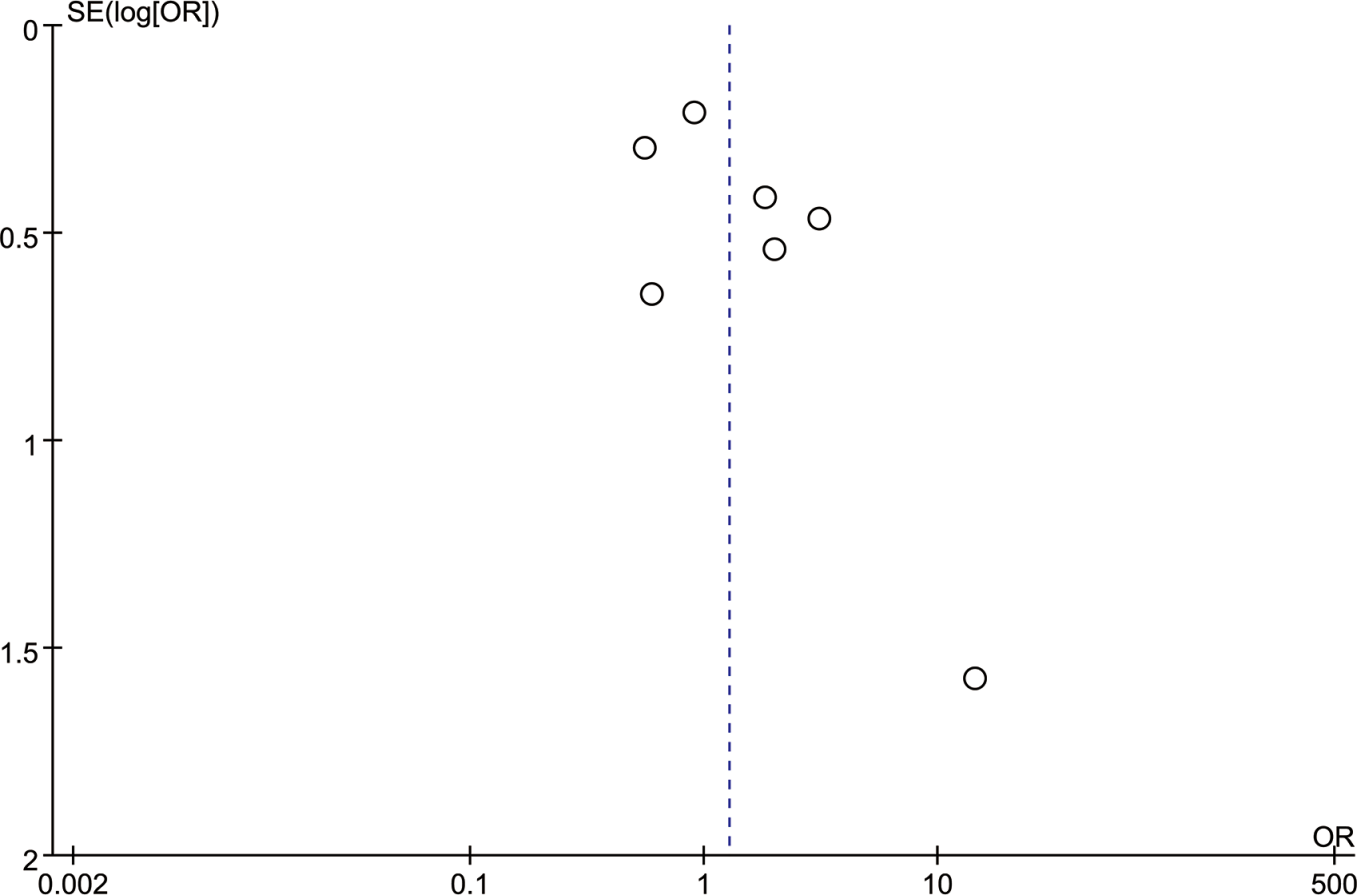
Funnel plot of BI-RADS score.

**Figure 5** is the funnel plot of the BI-RADS scores of all seven studies. Except for 1 study, there was rough symmetry between the two sides, indicating that there was a certain bias in the statistical results, but the bias was not large (20). To further evaluate publication bias, eggers tests were performed, which showed no significant publication bias (P=0.170).

**Figure 6:**
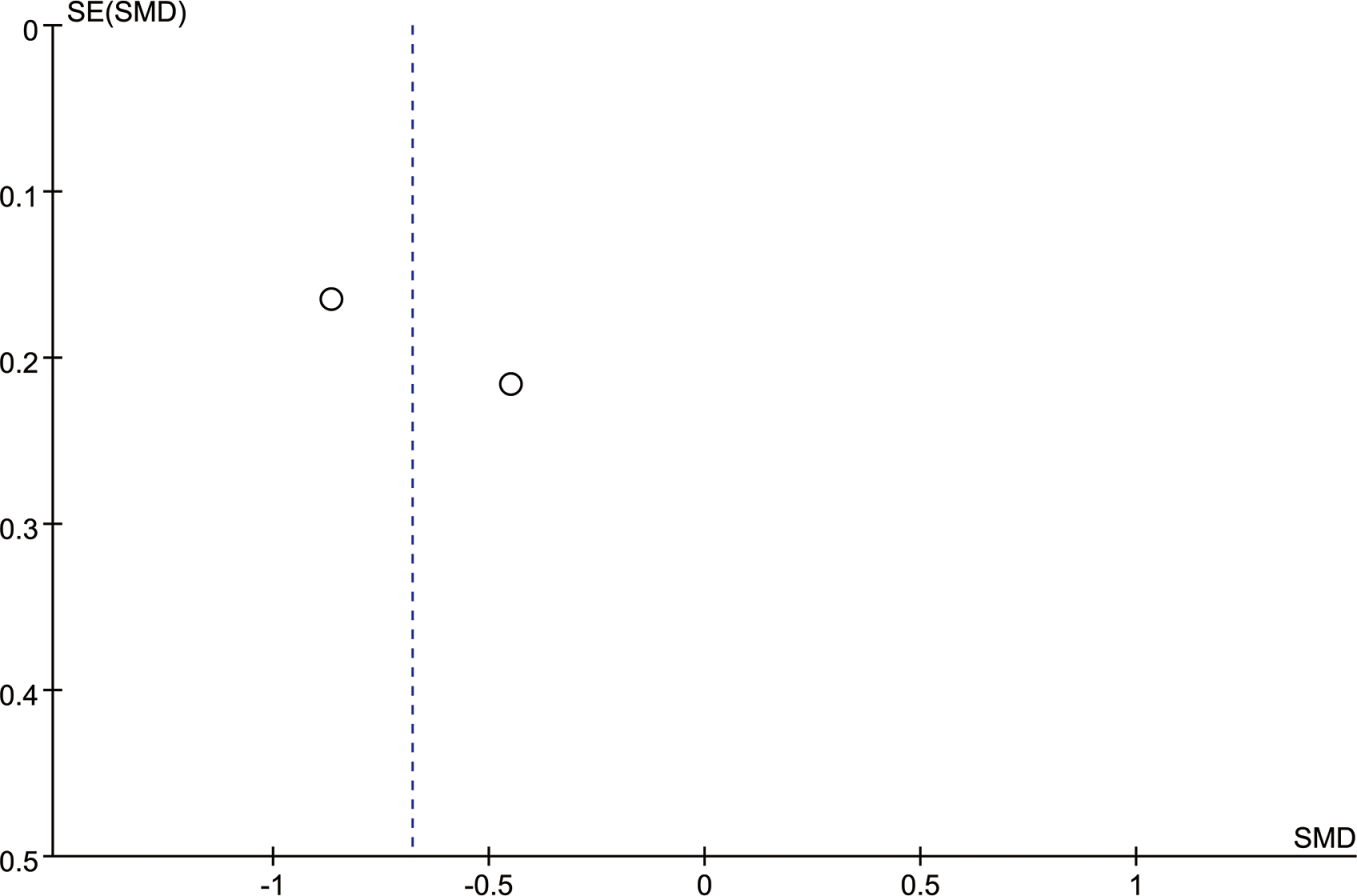
Funnel plot of VDG score.

**Figure 6** shows the funnel plot of the two studies that reported VDG scores. The two sides were roughly symmetrical, so there was no bias (20).

## Discussion

Our study included 535 people and used qualitative breast densitometry (BI-RADS) and quantitative breast densitometry (LIBRA and Volpara). The results of the meta-analysis showed that after bariatric surgery, VBD increased (P = 0.001), while BI-RADS grade A decreased by 3.83% (183 vs. 176) (P = 0.38). However, methods for quantifying breast density from before to after bariatric surgery by other software all yielded positive results (15, 17). This discrepancy in results was due to differences in breast density evaluation software and evaluation methods. The qualitative breast densitometry of BI-RADS refers to the radiologist’s grading of the breast based on the proportion of dense tissue areas on radiographs (6), but this classification method, based on the radiologist’s visual inspection, has a certain degree of subjectivity and wide variability (21). In contrast with the qualitative evaluation of BI-RADS, Volpara’s quantitative breast density is calculated from total breast volume and fibroglandular tissue volume in three dimensions, and VBD is calculated through a specific algorithm (9). Its accuracy and repeatability are better than those of the qualitative measurement through BI-RADS (22).

Breast density is affected by many factors, such as age, BMI, diet, lifestyle, chronic inflammation, medications, and reproductive history (23–32). Breast density declines with age begin before menopause, continue after menopause, and decline is most pronounced during the menopausal transition (23). People who ate a diet high in protein, carbohydrates and meat had higher breast density, while fat and vitamin intake had no effect on breast density. Some studies have shown that carotenoids and fibre can also reduce breast density (24, 25). Poor lifestyle habits such as alcohol consumption can increase breast density, while smoking can decrease breast density (26, 27). For a woman’s reproductive history, breast density was higher in women who did not give birth, and lower in women who gave birth early and often (30).In addition, both obesity and dense breasts are important risk factors for breast cancer (33), but obese patients have lower breast density, so we cannot simply use breast density to evaluate the risk of breast cancer in obese people at this time (32). Mechanistically, these factors may affect breast density by affecting oestrogen production, aromatase activity, and the growth hormone–insulin-like growth factor axis (23–32, 34).

Mammography plays an irreplaceable role in the clinical detection of changes in breast density and early breast cancer. From early screen-film mammography to digital mammography (full-field digital mammography) to the emerging technology of digital breast tomosynthesis (DBT), imaging has improved from two-dimensional to three-dimensional, and image quality has been continuously improved (35–37) DBT is a 3D image set reconstructed by collecting 2D raw projection images within a certain angle on an arc. Unlike full-field digital mammography and screen-film mammography, DBT quantifies dense breast tissue out of a volume of 3D space, resulting in more accurate identification and differentiation between dense and adipose tissue (38, 39), while the determination of breast density is based on mammography. Breast densitometry can be divided into qualitative and quantitative methods. Martin et al. (2006) (40) have shown that there is a significant difference between qualitative and quantitative measurement results. Morrish et al. (2015) (41) showed that compared with qualitative breast density, quantitative breast density can more accurately reflect changes in breast density. Density measurement methods are divided into breast-based classification methods, such as BI-RADS; area-based measurement methods, such as LIBRA and Cumulus; and volume-based measurement methods, such as Quantra and Volpara (42). BI-RADS is currently the most widely used clinically, but it is subjective (21). The area-based classification method measures breast density from the perspective of two-dimensional space, it cannot capture the volume of dense tissue, and it ignores the effect of tissue thickness on breast density, so it is inaccurate for breast density calculation (43, 44). The volume-based measurement methods measure breast density from the perspective of three-dimensional space volume, and its results are more accurate. The results obtained by different breast densitometry methods are quite different. The differences are as high as 14% in the classification of women with dense breasts (45). Alonzo et al. (2015) (46) and Duffy et al. (2018) (47) showed that Volpara is the most reliable, as it can more accurately reflect the changes in breast density than other breast density measurement methods. Lovrics et al. (2021) (48) showed that the increase in breast density and the decrease in breast volume after bariatric surgery respectively increased and decreased the effectiveness of mammography, which may ultimately make the results of mammography unchanged. This study ignored changes in breast parenchymal composition after bariatric surgery, and the reduction of breast volume may not offset the effect of changes in parenchymal components such as fibrous glands on mammographic performance. As the number of breast cancer patients increase, finding a way to accurately reflect changes in breast density becomes critical. Gastounioti et al. (2021) (49) combined DBT with Volpara to obtain excellent results. It could be that with the popularity and price decline of DBT and Volpara, clinicians will more accurately capture subtle changes in breast tissue and better predict breast cancer risk.

### Limitations of this review

First, our study was retrospective. Second, in our study, Caucasians were predominant, followed by Black people and Asian races, and the changes in breast density from before to after bariatric surgery in different races may affect our results. We plan to carry out a series of studies in Asian populations in the future. Last, since our study did not clarify the changes in breast density of different bariatric surgery methods, the results obtained by different surgical methods may also be different. In the future, with the gradual unification of surgical methods, this problem should be solved.

## Conclusion

Regarding the changes in breast density from before to after bariatric surgery, the volumetric VDG score increased after bariatric surgery, but there was no significant change in breast density by the BI-RADS score. Overall, breast density increased significantly in patients after bariatric surgery, but this depended on the method of breast densitometry. More randomized controlled studies are needed to validate our conclusions.

## Data availability statement

The datasets presented in this study can be found in online repositories. The names of the repository/repositories and accession number(s) can be found in the article/**Supplementary Material**.

## Author contributions

YS and CL designed the research; DS conducted the research; DS, ZH, and WD conducted data analysis; DS drafted the manuscript; CL, ZH, and XZ made revisions to the manuscript. All authors contributed to the article and approved the submitted version.
