# Supplementary material for: Effects of bariatric surgery on breast density in adult obese women: systematic review and meta-analysis
Source: Front Immunol. 2023 May 31;14:1160809. doi: 10.3389/fimmu.2023.1160809 (PMC10264659; doi:10.3389/fimmu.2023.1160809)
Supplement: Supplementary file 1 [file Table_1.docx]

**Effects of bariatric surgery on breast density in adult obese women: Systematic review and meta-analysis**

Dezheng Sun ^1†^, Zhiping Huang ^2†^, Wenyan Dong ^1^, Xiang Zhao ^1^, Chaoqian Liu^1 *^, Yuan Sheng ^1 *^

***** Corresponding authors

Chaoqian Liu [liuchaoqian2002@aliyun.com](mailto:liuchaoqian2002@aliyun.com), Yuan Sheng <sheng528yuan@163.com>

1. Search strategies

(1)The search statement of Pubmed

| Number | Queries | Results |
| --- | --- | --- |
| #1 | ductal[Title/Abstract]ORmammar*[Title/Abstract]OR breast*[Title/Abstract]ORbreast[MeSH Terms] | 631433 |
| #2 | Bariatric Surgery[MeSH Terms]  OR bariatric surger*[Title/Abstract]  OR stomach stapling[Title/Abstract]  OR duodenal switch[Title/Abstract]  OR sleeve gastrectomy[Title/Abstract]  OR gastric banding[Title/Abstract]  OR jejunoileal bypass[Title/Abstract]  OR biliopancreatic bypass[Title/Abstract]  OR biliopancreatic diversion[Title/Abstract]  OR Roux-en-Y[Title/Abstract]  OR gastric bypass[Title/Abstract]  OR stomach bypass[Title/Abstract]  OR gastroplasty[Title/Abstract]  OR metabolic surger*[Title/Abstract]  OR weight loss surger*[Title/Abstract] | 49202 |
| #3 | #1 and #2 | 813 |

(2）The search statement of Embase

| Number | Queries | Results |
| --- | --- | --- |
| #1 | breast*****:ab,ti OR mammar*****:ab,ti OR ductal:ab,tiOR breast/exp | 872876 |
| #2 | 'bariatric surger*':ab,ti  OR 'metabolic surger*':ab,ti  OR gastroplasty:ab,ti  OR 'stomach bypass':ab,ti  OR 'gastric bypass':ab,ti  OR 'roux en y':ab,ti  OR 'biliopancreatic diversion':ab,ti  OR 'biliopancreatic bypass':ab,ti  OR 'jejunoileal bypass':ab,ti  OR 'gastric banding':ab,ti  OR 'sleeve gastrectomy':ab,ti  OR 'duodenal switch':ab,ti  OR 'stomach stapling':ab,ti  OR 'weight loss surger*':ab,ti  OR'bariatric surgical procedure*':ab,ti  OR 'bariatric surgery'/exp | 81886 |
| #3 | #1 and #2 | 738 |

2.Exclude studies after reading the full text

| Title | Exclusion of cause |
| --- | --- |
| Obesity Surgery and Cancer: What Are the Unanswered Questions? | No preoperative and postoperative X-ray |
| Simple Reshaping of the Breast in Massive Weight Loss Patients | No preoperative and postoperative X-ray |
| Reduction Mammaplasty, Obesity, and Massive Weight Loss: Temporal Relationships of Satisfaction with Breast Contour | No preoperative and postoperative X-ray |
| Benign, Premalignant, and Malignant Lesions Encountered in Bariatric Surgery | No preoperative and postoperative X-ray |
| Autologous breast reconstruction in the postbariatric patient population | No preoperative and postoperative X-ray |
| Characterizing Breast Deformities After Massive Weight Loss | No preoperative and postoperative X-ray |
| 3D Mammometric Changes in the Treatment of Idiopathic  Gynecomastia | Male breast |
| Breast Contouring in Postbariatric Patients: A Technique Selection Algorithm | No preoperative and postoperative X-ray |
| Early experience with lightweight breast implants in breast reconstruction and massive weight loss patients | No preoperative and postoperative X-ray |
| PATIENT DOSE DURING RADIOLOGICAL EXAMINATION IN THE FOLLOW-UP OF BARIATRIC SURGERY | Only the dose of X-ray |
| Psychosocial functioning and quality of life in patients with loose redundant skin 4 to 5 years after bariatric surgery | No preoperative and postoperative X-ray |
| Breast Density Changes with Weight Gain/Loss after Bariatric Surgery | Conference abstract |
| Breast density increases after bariatric surgery | Conference abstract |
| Desire for Body Contouring Surgery After Laparoscopic Sleeve Gastrectomy | No preoperative and postoperative X-ray |
| Breast Reshaping Following Bariatric Surgery | No preoperative and postoperative X-ray |
| The Short-Term Effect of Weight Loss Surgery on Breast Density | Conference abstract |
| The Prevalence of Body Contouring Surgery After Gastric Bypass Surgery | No preoperative and postoperative X-ray |
| Autologous Augmentation-Mastopexy After Bariatric Surgery: Waste Not Want Not! | No preoperative and postoperative X-ray |
| The Impact of Reconstructive Procedures Following Bariatric Surgery on Patient Well-being and Quality of Life | No preoperative and postoperative X-ray |
| Psychosocial Consequences of Weight Loss following Gastric Banding for Morbid Obesity | No preoperative and postoperative X-ray |
| Breast Reconstruction Facilitated by Vertical Banded Gastroplasty | No preoperative and postoperative X-ray |
| The Impact of Bariatric Surgery on Breast Cancer Recurrence: Case Series and Review of Literature | No preoperative and postoperative X-ray |
| Obesity and Cancer: the Profile of a Population who Underwent Bariatric Surgery | No preoperative and postoperative X-ray |
| Bariatric Surgery and Breast Cancer Incidence: a Population‑Based, Matched Cohort Study | No preoperative and postoperative X-ray |
| Value of magnetic resonance diffusion combined with perfusion imaging techniques for diagnosing potentially malignant breast lesions | No preoperative and postoperative X-ray |
| Bariatric surgery could reduce the risk of specific types of breast cancer | No preoperative and postoperative X-ray |
| Bariatric Surgery Does Not Appear to Affect Women’s Breast-Milk Composition | No preoperative and postoperative X-ray |
| Body contouring following massive weight loss | No preoperative and postoperative X-ray |
| Clinical characteristics and treatment of chest submammary accessory breasts | No preoperative and postoperative X-ray |
| Gynécomastia. Management of diagnosis and therapy. Apropos of 148 cases | Male breast |
| Contouring of the male anterior chest following bariatric surgery and massive weight loss | Male breast |
| Breast reshaping after massive weight loss | No preoperative and postoperative X-ray |
| Aspects of excess skin in obesity, after weight loss, after body contouring surgery and in a reference population | No preoperative and postoperative X-ray |
| Women's experiences of pregnancy and lactation after bariatric surgery: A scoping review | No preoperative and postoperative X-ray |
| Breast reduction outcome study | No preoperative and postoperative X-ray |
| Reduction mammoplasty techniques in post-bariatric patients: our experience | No preoperative and postoperative X-ray |
| Vertical Mastopexy and Lateral Intercostal Artery Perforator (LICAP) Flap With Pectoralis Muscle Sling for Autologous Tissue Breast Augmentation in the Bariatric Patient | No preoperative and postoperative X-ray |
| Mammary Volume-to-Body Mass Index Ratio: Preoperative Predictor  of Cardiovascular Risk Factor and Indicator of Long-term Postoperative Remission of Comorbidities in Premenopausal Women Undergoing Sleeve Gastrectomy | Only preoperative X-ray |

3.The results of meta-regression analysis

| concomitant variable | P |
| --- | --- |
| Mean time from preoperative mammogram to postoperative mammogram (year) | 0.635 |
| RYGB | 0.018 |
| SG | 0.435 |
| LAGB | 0.541 |
| the average decrease in BMI( kg/m2) | 0.080 |
| published time(year) | 0.741 |
